# 2-Amino-5-methyl­pyridinium 4-chloro­benzoate

**DOI:** 10.1107/S1600536812051021

**Published:** 2012-12-22

**Authors:** Kaliyaperumal Thanigaimani, Abbas Farhadikoutenaei, Suhana Arshad, Ibrahim Abdul Razak

**Affiliations:** a School of Physics, Universiti Sains Malaysia, 11800 USM, Penang, Malaysia; b Department of Physics, Faculty of Science, University of Mazandaran, Babolsar, Iran

## Abstract

The 4-chloro­benzoate anion of the title salt, C_6_H_9_N_2_^+^·C_7_H_4_ClO_2_^−^, is nearly planar with a dihedral angle of 5.14 (16)° between the benzene ring and the carboxyl­ate group. In the crystal, the protonated N atom and the 2-amino group of the cation are hydrogen bonded to the carboxyl­ate O atoms of the anion *via* a pair of N—H⋯O hydrogen bonds with an *R*_2_^2^(8) ring motif. The ion pairs are further connected *via* N—H⋯O and weak C—H⋯O hydrogen bonds, forming a two-dimensional network parallel to the *bc* plane. The crystal structure also features a π–π stacking inter­action between the pyridinium and benzene rings with a centroid–centroid distance of 3.7948 (9) Å.

## Related literature

For background to the chemistry of substituted pyridines, see: Pozharski *et al.* (1997 ▶); Katritzky *et al.* (1996 ▶). For 4-chloro­benzoic acid, see: Dionysiou *et al.* (2000 ▶). For details of hydrogen-bonded supra­molecular compounds, see: Aakeroy *et al.* (2002 ▶). For related structures, see: Nahringbauer & Kvick (1977 ▶); Thanigaimani *et al.* (2012*a* ▶,*b* ▶,*c* ▶). For hydrogen-bond motifs, see: Bernstein *et al.* (1995 ▶). For bond-length data, see: Allen *et al.* (1987 ▶).
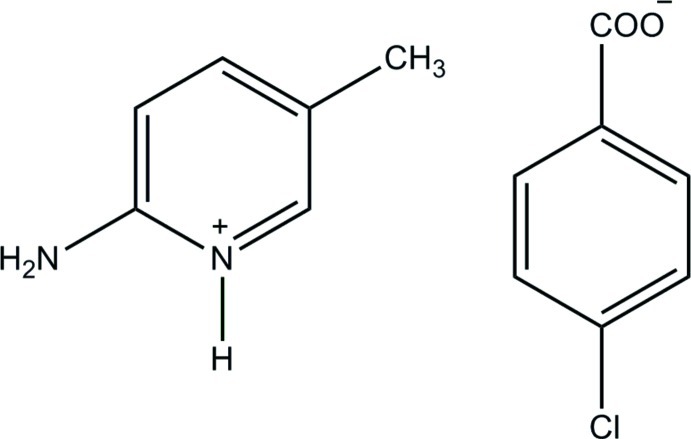


## Experimental

### 

#### Crystal data

C_6_H_9_N_2_^+^·C_7_H_4_ClO_2_^−^
*M* *_r_* = 264.70
Monoclinic, 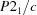

*a* = 9.8510 (6) Å
*b* = 10.7707 (8) Å
*c* = 12.2123 (7) Å
β = 102.335 (2)°
*V* = 1265.84 (14) Å^3^
*Z* = 4
Mo *K*α radiation
μ = 0.30 mm^−1^
*T* = 297 K
0.46 × 0.25 × 0.13 mm

#### Data collection

Bruker SMART APEXII DUO CCD area-detector diffractometer
Absorption correction: multi-scan (*SADABS*; Bruker, 2009 ▶) *T*_min_ = 0.876, *T*_max_ = 0.963
17572 measured reflections
4628 independent reflections
2976 reflections with *I* > 2σ(*I*)
*R*_int_ = 0.034

#### Refinement

*R*[*F* ^2^ > 2σ(*F* ^2^)] = 0.047
*wR*(*F* ^2^) = 0.139
*S* = 1.04
4628 reflections
176 parameters
H atoms treated by a mixture of independent and constrained refinement
Δρ_max_ = 0.30 e Å^−3^
Δρ_min_ = −0.31 e Å^−3^

### 

Data collection: *APEX2* (Bruker, 2009 ▶); cell refinement: *SAINT* (Bruker, 2009 ▶); data reduction: *SAINT*; program(s) used to solve structure: *SHELXTL* (Sheldrick, 2008 ▶); program(s) used to refine structure: *SHELXTL*; molecular graphics: *SHELXTL*; software used to prepare material for publication: *SHELXTL* and *PLATON* (Spek, 2009 ▶).

## Supplementary Material

Crystal structure: contains datablock(s) global, I. DOI: 10.1107/S1600536812051021/is5231sup1.cif

Structure factors: contains datablock(s) I. DOI: 10.1107/S1600536812051021/is5231Isup2.hkl

Supplementary material file. DOI: 10.1107/S1600536812051021/is5231Isup3.cml

Additional supplementary materials:  crystallographic information; 3D view; checkCIF report

## Figures and Tables

**Table 1:** Hydrogen-bond geometry (Å, °)

*D*—H⋯*A*	*D*—H	H⋯*A*	*D*⋯*A*	*D*—H⋯*A*
N2—H1*N*2⋯O1	0.85 (2)	2.02 (2)	2.8654 (18)	174.1 (19)
N1—H1*N*1⋯O1^i^	0.98 (2)	1.75 (2)	2.7255 (14)	174.1 (16)
N2—H2*N*2⋯O2^i^	0.92 (2)	1.83 (2)	2.7437 (17)	172.7 (18)
C2—H2*A*⋯O2^ii^	0.93	2.40	3.1459 (18)	137

Symmetry codes: (i) 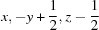; (ii) 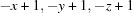.

**Fig. 1.**
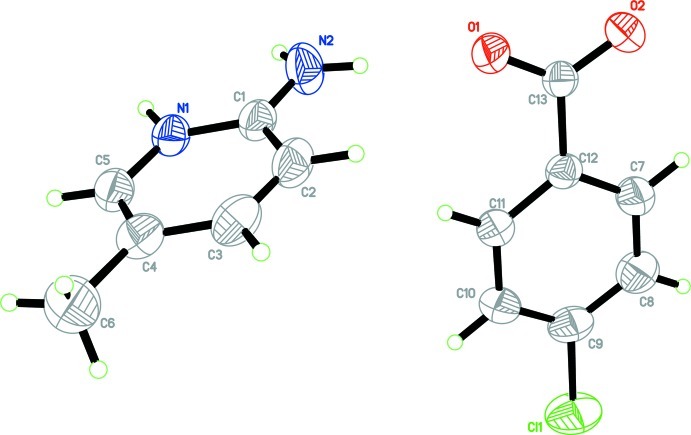
The molecular structure of the title compound with atom labels with 50% probability displacement ellipsoids.

**Fig. 2.**
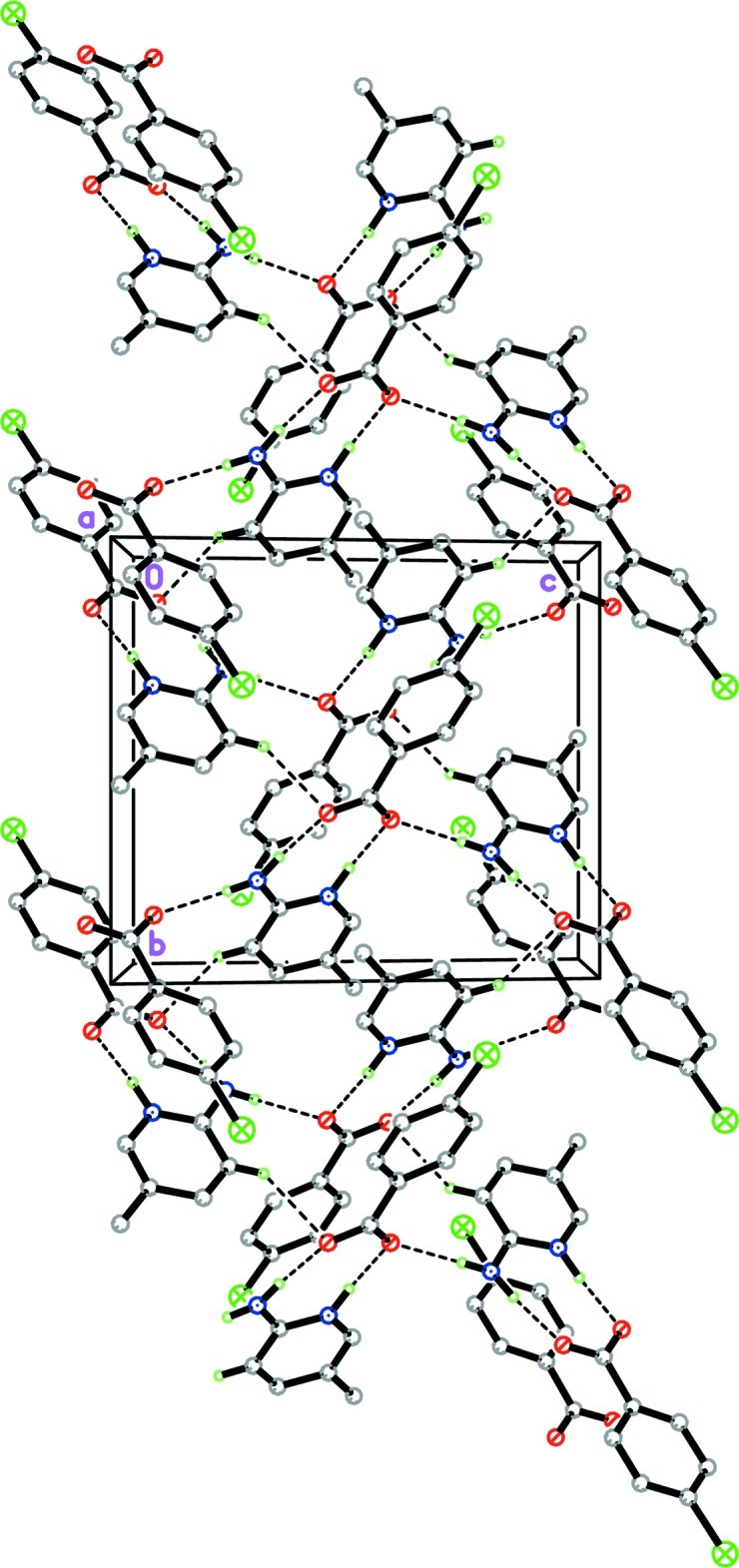
The crystal packing of the title compound. The H atoms not involved in the intermolecular interactions (dashed lines) have been omitted for clarity.

## supplementary crystallographic information

### Comment

In recent years, hydrogen bonds have attracted the interest of chemists and have been widely used to design and synthesize one, two and three-dimensional supramolecular compounds (Aakeroy *et al.*, 2002). Pyridine and its derivatives play an important role in heterocyclic chemistry (Pozharski *et al.*, 1997; Katritzky *et al.*, 1996). They are often involved in hydrogen-bond interactions. 4-Chlorobenzoic acid (4-CBA) has been reported as intermediate product during biological or chemical degradation of pesticides and herbicides (Dionysiou *et al.*, 2000). We have recently reported related crystal structures of 2-amino-5-methylpyridinium 3-chlorobenzoate (Thanigaimani *et al.*, 2012*a*), 2-amino-5-methylpyridinium 2-aminobenzoate (Thanigaimani *et al.*, 2012*b*) and 2-amino-5-methylpyridinium trifluoroacetate (Thanigaimani *et al.*, 2012*c*). In order to study some interesting hydrogen bonding interactions of this compound, the synthesis and structure of the title salt is presented here.

The asymmetric unit (Fig. 1) contains one 2-amino-5-methylpyridinium cation and one 4-chlorobenzoate anion. The proton transfers from the carboxyl group oxygen atom (O1) to atom N1 of the 2-amino-5-methylpyrimidine resulted in the widening of C1—N1—C5 angle of the pyridinium ring to 122.06 (11)°, compared to the corresponding angle of 117.4 (3)° in neutral 2-amino-5-methylpyridine (Nahringbauer & Kvick, 1977). The 2-amino-5-methylpyridinium cation is essentially planar, with a maximum deviation of 0.005 (1) Å for atom C1. The carboxylate group of the 4-chlorobenzoate anion is slightly twisted from the attached ring with the dihedral angle between C7–C12 ring and O1/O2/C13 plane being 5.14 (16)°. The bond lengths (Allen *et al.*, 1987) and angles are normal.

In the crystal packing (Fig. 2), the protonated N1 atom and a nitrogen atom of the 2-amino group (N2) are hydrogen-bonded to the carboxylate oxygen atoms (O1 and O2) *via* a pair of intermolecular N1—H1N1···O1^i^ and N2—H2N2···O2^i^ hydrogen bonds (symmetry code in Table 1), forming a ring motif of *R*_2_^2^(8) (Bernstein *et al.*, 1995). Furthermore, these motifs are connected *via* N2—H1N2···O1 and C2—H2A···O2^ii^ hydrogen bonds to form a two-dimensional network parallel to the *bc* plane. The crystal structure is further stabilized by π–π interactions between the pyridine (C1–C5/N1) and benzene (C7–C12) rings, with centroid to centroid distance of 3.7948 (9) Å (symmetry codes: 1 - *x*, -1/2 + *y*, 1/2 - *z* and 1 - *x*, 1/2 + *y*, 1/2 - *z*)

### Experimental

Hot methanol solutions (20 ml) of 2-amino-5-methylpyridine (54 mg, Aldrich) and 4-chlorobenzoic acid (39 mg, Aldrich) were mixed and warmed over a heating magnetic stirrer hotplate for a few minutes. The resulting solution was allowed to cool slowly at room temperature and crystals of the title compound (I) appeared after a few days.

### Refinement

N-bound H Atoms were located in a difference Fourier maps and refined freely [refined N—H distances 0.98 (2), 0.92 (2) and 0.85 (2) Å]. The remaining H atoms were positioned geometrically (C—H = 0.93–0.96 Å) and were refined using a riding model, with *U*_iso_(H) = 1.2*U*_eq_(C) or 1.5*U*_eq_(methyl C). A rotating group model was used for the methyl group. Three outliers were omitted (1 4 0, 1 1 0 and 3 1 2) in the final refinement.

### Crystal data

**Table:**

C_6_H_9_N_2_^+^·C_7_H_4_ClO_2_^−^	*F*(000) = 552
*M**_r_* = 264.70	*D*_x_ = 1.389 Mg m^−^^3^
Monoclinic, *P*2_1_/*c*	Mo *K*α radiation, λ = 0.71073 Å
Hall symbol: -P 2ybc	Cell parameters from 3497 reflections
*a* = 9.8510 (6) Å	θ = 2.6–26.9°
*b* = 10.7707 (8) Å	µ = 0.30 mm^−^^1^
*c* = 12.2123 (7) Å	*T* = 297 K
β = 102.335 (2)°	Plate, colourless
*V* = 1265.84 (14) Å^3^	0.46 × 0.25 × 0.13 mm
*Z* = 4	

### Data collection

**Table:**

Bruker SMART APEXII DUO CCD area-detector diffractometer	4628 independent reflections
Radiation source: fine-focus sealed tube	2976 reflections with *I* > 2σ(*I*)
Graphite monochromator	*R*_int_ = 0.034
φ and ω scans	θ_max_ = 32.8°, θ_min_ = 2.6°
Absorption correction: multi-scan (*SADABS*; Bruker, 2009)	*h* = −14→15
*T*_min_ = 0.876, *T*_max_ = 0.963	*k* = −16→16
17572 measured reflections	*l* = −18→17

### Refinement

**Table:**

Refinement on *F*^2^	Primary atom site location: structure-invariant direct methods
Least-squares matrix: full	Secondary atom site location: difference Fourier map
*R*[*F*^2^ > 2σ(*F*^2^)] = 0.047	Hydrogen site location: inferred from neighbouring sites
*wR*(*F*^2^) = 0.139	H atoms treated by a mixture of independent and constrained refinement
*S* = 1.04	*w* = 1/[σ^2^(*F*_o_^2^) + (0.0608*P*)^2^ + 0.1513*P*] where *P* = (*F*_o_^2^ + 2*F*_c_^2^)/3
4628 reflections	(Δ/σ)_max_ < 0.001
176 parameters	Δρ_max_ = 0.30 e Å^−^^3^
0 restraints	Δρ_min_ = −0.31 e Å^−^^3^

### Special details

**Table:**

Geometry. All e.s.d.'s (except the e.s.d. in the dihedral angle between two l.s. planes) are estimated using the full covariance matrix. The cell e.s.d.'s are taken into account individually in the estimation of e.s.d.'s in distances, angles and torsion angles; correlations between e.s.d.'s in cell parameters are only used when they are defined by crystal symmetry. An approximate (isotropic) treatment of cell e.s.d.'s is used for estimating e.s.d.'s involving l.s. planes.
Refinement. Refinement of *F*^2^ against ALL reflections. The weighted *R*-factor *wR* and goodness of fit *S* are based on *F*^2^, conventional *R*-factors *R* are based on *F*, with *F* set to zero for negative *F*^2^. The threshold expression of *F*^2^ > σ(*F*^2^) is used only for calculating *R*-factors(gt) *etc*. and is not relevant to the choice of reflections for refinement. *R*-factors based on *F*^2^ are statistically about twice as large as those based on *F*, and *R*- factors based on ALL data will be even larger.

### Fractional atomic coordinates and isotropic or equivalent isotropic displacement parameters (Å^2^)

**Table:**

	*x*	*y*	*z*	*U*_iso_*/*U*_eq_	
Cl1	−0.02173 (5)	0.82984 (4)	0.23669 (4)	0.07093 (17)	
O1	0.41482 (11)	0.36071 (10)	0.43150 (8)	0.0468 (2)	
O2	0.29316 (12)	0.37788 (10)	0.56405 (8)	0.0521 (3)	
N1	0.54982 (12)	0.32374 (10)	0.06443 (9)	0.0384 (2)	
N2	0.44920 (16)	0.28252 (14)	0.21480 (12)	0.0534 (3)	
C1	0.53584 (14)	0.34947 (12)	0.16960 (11)	0.0393 (3)	
C2	0.61705 (15)	0.44688 (14)	0.22692 (12)	0.0462 (3)	
H2A	0.6104	0.4672	0.2996	0.055*	
C3	0.70468 (15)	0.51067 (14)	0.17600 (13)	0.0490 (3)	
H3A	0.7581	0.5743	0.2148	0.059*	
C4	0.71723 (14)	0.48309 (13)	0.06545 (12)	0.0452 (3)	
C5	0.63774 (14)	0.38868 (13)	0.01390 (11)	0.0416 (3)	
H5A	0.6435	0.3675	−0.0588	0.050*	
C6	0.81460 (18)	0.55543 (18)	0.01020 (17)	0.0643 (4)	
H6A	0.8137	0.5209	−0.0624	0.096*	
H6B	0.9070	0.5510	0.0555	0.096*	
H6C	0.7853	0.6406	0.0022	0.096*	
C7	0.14475 (15)	0.57790 (14)	0.46421 (12)	0.0449 (3)	
H7A	0.1323	0.5510	0.5338	0.054*	
C8	0.06266 (16)	0.67279 (15)	0.40995 (14)	0.0530 (4)	
H8A	−0.0053	0.7091	0.4420	0.064*	
C9	0.08322 (14)	0.71276 (13)	0.30744 (12)	0.0452 (3)	
C10	0.18420 (16)	0.66134 (13)	0.25949 (12)	0.0452 (3)	
H10A	0.1978	0.6902	0.1909	0.054*	
C11	0.26556 (14)	0.56587 (12)	0.31472 (11)	0.0399 (3)	
H11A	0.3344	0.5309	0.2830	0.048*	
C12	0.24526 (12)	0.52207 (11)	0.41692 (10)	0.0336 (2)	
C13	0.32454 (13)	0.41235 (11)	0.47546 (10)	0.0356 (3)	
H1N1	0.4981 (19)	0.2561 (18)	0.0206 (15)	0.067 (5)*	
H2N2	0.393 (2)	0.227 (2)	0.1687 (17)	0.075 (6)*	
H1N2	0.4328 (19)	0.3062 (17)	0.2770 (17)	0.060 (5)*	

### Atomic displacement parameters (Å^2^)

**Table:**

	*U*^11^	*U*^22^	*U*^33^	*U*^12^	*U*^13^	*U*^23^
Cl1	0.0665 (3)	0.0571 (3)	0.0817 (3)	0.02362 (19)	−0.0011 (2)	0.0172 (2)
O1	0.0589 (6)	0.0468 (5)	0.0353 (5)	0.0185 (4)	0.0114 (4)	0.0030 (4)
O2	0.0713 (7)	0.0503 (6)	0.0373 (5)	0.0150 (5)	0.0171 (5)	0.0093 (4)
N1	0.0453 (6)	0.0363 (5)	0.0330 (5)	0.0000 (4)	0.0069 (4)	−0.0063 (4)
N2	0.0695 (8)	0.0554 (8)	0.0388 (6)	−0.0138 (7)	0.0196 (6)	−0.0123 (6)
C1	0.0468 (7)	0.0369 (6)	0.0332 (6)	0.0043 (5)	0.0063 (5)	−0.0056 (5)
C2	0.0529 (8)	0.0457 (7)	0.0384 (7)	0.0010 (6)	0.0060 (6)	−0.0134 (6)
C3	0.0460 (7)	0.0418 (7)	0.0564 (8)	−0.0020 (6)	0.0050 (6)	−0.0140 (6)
C4	0.0424 (7)	0.0400 (7)	0.0532 (8)	0.0028 (5)	0.0102 (6)	−0.0018 (6)
C5	0.0457 (7)	0.0415 (7)	0.0380 (6)	0.0048 (5)	0.0101 (5)	−0.0026 (5)
C6	0.0588 (9)	0.0611 (10)	0.0768 (12)	−0.0101 (8)	0.0231 (8)	−0.0026 (9)
C7	0.0498 (7)	0.0446 (7)	0.0428 (7)	0.0064 (6)	0.0157 (6)	0.0043 (6)
C8	0.0503 (8)	0.0513 (8)	0.0608 (9)	0.0148 (6)	0.0195 (7)	0.0036 (7)
C9	0.0414 (7)	0.0365 (6)	0.0533 (8)	0.0052 (5)	−0.0001 (6)	0.0030 (6)
C10	0.0534 (8)	0.0405 (7)	0.0408 (7)	0.0040 (6)	0.0083 (6)	0.0073 (5)
C11	0.0442 (6)	0.0379 (6)	0.0389 (6)	0.0053 (5)	0.0117 (5)	0.0023 (5)
C12	0.0365 (6)	0.0295 (5)	0.0330 (6)	−0.0017 (4)	0.0036 (4)	−0.0022 (4)
C13	0.0453 (6)	0.0314 (6)	0.0280 (5)	0.0013 (5)	0.0029 (5)	−0.0029 (4)

### Geometric parameters (Å, º)

**Table:**

Cl1—C9	1.7390 (14)	C5—H5A	0.9300
O1—C13	1.2619 (16)	C6—H6A	0.9600
O2—C13	1.2436 (16)	C6—H6B	0.9600
N1—C1	1.3495 (16)	C6—H6C	0.9600
N1—C5	1.3596 (18)	C7—C8	1.382 (2)
N1—H1N1	0.98 (2)	C7—C12	1.3856 (18)
N2—C1	1.3245 (19)	C7—H7A	0.9300
N2—H2N2	0.92 (2)	C8—C9	1.379 (2)
N2—H1N2	0.85 (2)	C8—H8A	0.9300
C1—C2	1.4113 (19)	C9—C10	1.374 (2)
C2—C3	1.354 (2)	C10—C11	1.3870 (18)
C2—H2A	0.9300	C10—H10A	0.9300
C3—C4	1.413 (2)	C11—C12	1.3881 (18)
C3—H3A	0.9300	C11—H11A	0.9300
C4—C5	1.3539 (19)	C12—C13	1.5097 (16)
C4—C6	1.503 (2)		
			
C1—N1—C5	122.05 (12)	C4—C6—H6C	109.5
C1—N1—H1N1	121.7 (10)	H6A—C6—H6C	109.5
C5—N1—H1N1	116.2 (10)	H6B—C6—H6C	109.5
C1—N2—H2N2	117.2 (12)	C8—C7—C12	121.22 (13)
C1—N2—H1N2	118.2 (13)	C8—C7—H7A	119.4
H2N2—N2—H1N2	122.3 (17)	C12—C7—H7A	119.4
N2—C1—N1	119.39 (13)	C9—C8—C7	118.80 (13)
N2—C1—C2	123.06 (13)	C9—C8—H8A	120.6
N1—C1—C2	117.55 (13)	C7—C8—H8A	120.6
C3—C2—C1	119.92 (13)	C10—C9—C8	121.41 (13)
C3—C2—H2A	120.0	C10—C9—Cl1	119.23 (12)
C1—C2—H2A	120.0	C8—C9—Cl1	119.36 (11)
C2—C3—C4	121.78 (13)	C9—C10—C11	119.16 (13)
C2—C3—H3A	119.1	C9—C10—H10A	120.4
C4—C3—H3A	119.1	C11—C10—H10A	120.4
C5—C4—C3	116.24 (13)	C10—C11—C12	120.66 (12)
C5—C4—C6	122.81 (14)	C10—C11—H11A	119.7
C3—C4—C6	120.95 (14)	C12—C11—H11A	119.7
C4—C5—N1	122.46 (13)	C7—C12—C11	118.71 (12)
C4—C5—H5A	118.8	C7—C12—C13	119.06 (11)
N1—C5—H5A	118.8	C11—C12—C13	122.18 (11)
C4—C6—H6A	109.5	O2—C13—O1	124.56 (12)
C4—C6—H6B	109.5	O2—C13—C12	116.49 (11)
H6A—C6—H6B	109.5	O1—C13—C12	118.93 (11)
			
C5—N1—C1—N2	−179.57 (13)	C7—C8—C9—Cl1	178.64 (12)
C5—N1—C1—C2	−0.37 (19)	C8—C9—C10—C11	1.0 (2)
N2—C1—C2—C3	179.28 (15)	Cl1—C9—C10—C11	−178.43 (11)
N1—C1—C2—C3	0.1 (2)	C9—C10—C11—C12	0.3 (2)
C1—C2—C3—C4	0.4 (2)	C8—C7—C12—C11	2.0 (2)
C2—C3—C4—C5	−0.6 (2)	C8—C7—C12—C13	−175.35 (13)
C2—C3—C4—C6	179.69 (15)	C10—C11—C12—C7	−1.8 (2)
C3—C4—C5—N1	0.3 (2)	C10—C11—C12—C13	175.48 (12)
C6—C4—C5—N1	−179.95 (14)	C7—C12—C13—O2	0.45 (17)
C1—N1—C5—C4	0.1 (2)	C11—C12—C13—O2	−176.81 (12)
C12—C7—C8—C9	−0.7 (2)	C7—C12—C13—O1	179.13 (12)
C7—C8—C9—C10	−0.8 (2)	C11—C12—C13—O1	1.87 (18)

### Hydrogen-bond geometry (Å, º)

**Table:**

*D*—H···*A*	*D*—H	H···*A*	*D*···*A*	*D*—H···*A*
N2—H1*N*2···O1	0.85 (2)	2.02 (2)	2.8654 (18)	174.1 (19)
N1—H1*N*1···O1^i^	0.98 (2)	1.75 (2)	2.7255 (14)	174.1 (16)
N2—H2*N*2···O2^i^	0.92 (2)	1.83 (2)	2.7437 (17)	172.7 (18)
C2—H2*A*···O2^ii^	0.93	2.40	3.1459 (18)	137

Symmetry codes: (i) *x*, −*y*+1/2, *z*−1/2; (ii) −*x*+1, −*y*+1, −*z*+1.
